# Ethyl pyruvate inhibits oxidation of LDL in vitro and attenuates oxLDL toxicity in EA.hy926 cells

**DOI:** 10.1371/journal.pone.0191477

**Published:** 2018-01-25

**Authors:** Christine Rossmann, Christoph Nusshold, Margret Paar, Gerhard Ledinski, Erwin Tafeit, Martin Koestenberger, Eva Maria Bernhart, Wolfgang Sattler, Gerhard Cvirn, Seth Hallström

**Affiliations:** 1 Institute of Physiological Chemistry, Medical University of Graz, Graz, Austria; 2 Department of Pediatrics, Medical University of Graz, Graz, Austria; 3 Institute of Molecular Biology and Biochemistry, Medical University of Graz, Graz, Austria; Qatar University College of Health Sciences, QATAR

## Abstract

**Background:**

Ethyl pyruvate (EP) exerts anti-inflammatory and anti-oxidative properties. The aim of our study was to investigate whether EP is capable of inhibiting the oxidation of LDL, a crucial step in atherogenesis. Additionally, we examined whether EP attenuates the cytotoxic effects of highly oxidized LDL in the human vascular endothelial cell line EA.hy926.

**Methods:**

Native LDL (nLDL) was oxidized using Cu^2+^ ions in the presence of increasing amounts of EP. The degree of LDL oxidation was quantified by measuring lipid hydroperoxide (LPO) and malondialdehyde (MDA) concentrations, relative electrophoretic mobilities (REMs), and oxidation-specific immune epitopes. The cytotoxicity of these oxLDLs on EA.hy926 cells was assessed by measuring cell viability and superoxide levels. Furthermore, the cytotoxicity of highly oxidized LDL on EA.hy926 cells under increasing concentrations of EP in the media was assessed including measurements of high energy phosphates (ATP).

**Results:**

Oxidation of nLDL using Cu^2+^ ions was remarkably inhibited by EP in a concentration-dependent manner, reflected by decreased levels of LPO, MDA, REM, oxidation-specific epitopes, and diminished cytotoxicity of the obtained oxLDLs in EA.hy926 cells. Furthermore, the cytotoxicity of highly oxidized LDL on EA.hy926 cells was remarkably attenuated by EP added to the media in a concentration-dependent manner reflected by a decrease in superoxide and an increase in viability and ATP levels.

**Conclusions:**

EP has the potential for an anti-atherosclerotic drug by attenuating both, the oxidation of LDL and the cytotoxic effect of (already formed) oxLDL in EA.hy926 cells. Chronic administration of EP might be beneficial to impede the development of atherosclerotic lesions.

## Introduction

Oxidation of low-density lipoprotein (LDL) is a central element in the development of atherosclerosis [1]. LDL in its native state (nLDL) is not atherogenic. However, in the subendothelial space of arterial sites, nLDL can become subject to oxidation by mechanisms involving free radicals and/or lipoxygenases [2]. The resulting oxidized form of nLDL, oxLDL, contains, i.a., malondialdehyde (MDA) and 4-hydroxynonenal (HNE), which have been shown to exert prominent cytotoxic effects on endothelial cells, a prerequisite for the pathogenesis of atherosclerosis [3, 4].

Presumably, drugs capable of suppressing oxidation of LDL possess anti-atherosclerotic properties. Ethyl pyruvate (EP) is such a candidate [5]. Antioxidant action of EP has already been shown in vivo using animal models [6]. For example, Tawadrous et al. have shown that EP is capable of suppressing lipid peroxidation: Treatment with EP attenuated hepatic MDA formation in rats subjected to oxidative stress [7].

It was the aim of our study to investigate whether EP is capable of suppressing the oxidation of LDL by using a well-established in vitro model. In the presence of increasing amounts of EP Cu^2+^ ions were used to mediate LDL oxidation. The degree of oxidation of the lipid part of the LDL particle was assessed by measuring lipid hydroperoxide (LPO) as well as MDA concentrations. Oxidation of the lipid part of LDL has been shown to be followed by modification of apolipoprotein B (apoB), the protein part of LDL [2]. We, therefore, also assessed the degree of apoB modification by measuring relative electrophoretic mobilities (REMs), and by quantifying oxidation-specific immune epitopes using a fluorescent immunoassay and specific antibodies against oxLDL [8, 9]. Furthermore, we assessed the cytotoxicity of oxLDL obtained by oxidation of nLDL in the presence of various amounts of EP. For this purpose human vascular endothelial EA.hy926 cells were incubated with the respective oxLDLs and cellular viability was examined by means of a standard test (3-(4,5-dimethyl-2-thiazolyl)-2,5-diphenyl-2H-tetrazolium bromide (MTT) assay, [10]). As a marker of oxidative stress, cellular superoxide levels were measured by high-performance liquid chromatography (HPLC) [11] using a method based on the reduction of dihydroethidium (DHE) to 2-hydroxyethidium by superoxide (O_2_^●─^). Moreover, mitochondrial function was monitored by measuring intracellular high energy phosphates using HPLC. Additionally, we investigated whether EP is capable of attenuating the cytotoxic effect of already oxidized LDL on endothelial cells. To test this hypothesis, EA.hy926 cells were incubated with highly oxidized LDL in the presence of increasing amounts of EP and the respective viabilities and superoxide and ATP levels were measured.

## Material and methods

### Preparation of LDL

The study was approved by the appropriate institutional review board (ethics committee of the Medical University of Graz; 27–320 ex 14/15) and written informed consent was obtained. Human LDL (1.020 to 1.063 g/mL) was obtained from the plasma of normolipemic (Lp(a) < 5 mg/dL), fasting (12 to 14 h) male donors (a total of 7 healthy volunteers aged between 29 and 44 years) by potassium bromide sequential ultracentrifugation [12]. Pefabloc (50 μM, Sigma-Aldrich, Vienna, Austria), butylated hydroxytoluene (20 μM, Sigma-Aldrich), and EDTA (1 g/L, Merck, Darmstadt, Germany) were present during all steps of lipoprotein preparation to prevent lipid peroxidation and apoB cleavage by contaminating bacteria or proteinases. The samples were sterile-filtered and stored at 4°C in the dark until use. The protein content of LDL was measured using the Lowry method [13]. Total cholesterol of the isolated LDL was determined enzymatically with the CHOD-iodide test kit (Boehringer-Mannheim, Germany).

### LDL oxidation using Cu ^2+^ ions

nLDL (1.5 mg/mL) was preincubated with various amounts of EP (0–1000 μg/mL) for 30 min at 37°C in 0.01 mol/L phosphate buffer (pH = 7.4) containing 0.154 mol/L NaCl. Subsequently, nLDL oxidation was initiated by addition of CuCl_2_ (10 μmol/L, final concentration) for up to 8 h. For cell experiments LDL oxidation was stopped when LPO levels reached 100 nmol/mg LDL which was followed by dialysis against PBS over night at 4°C.

### Determination of LPOs

The amount of LPO generated during LDL oxidation was determined with a spectrophotometric assay for lipid hydroperoxides in serum lipoproteins [14]. In principle, lipid peroxides are capable of converting iodide to iodine. Briefly, 50 μL of LDL-solution (containing 1.5 mg/mL of total LDL) was mixed on a vortex mixer with 500 μL of a colour reagent taken from the commercially available CHOD-PAP test kit. The samples were allowed to stand for 30 min at ambient temperature. The absorbance was measured at 365 nm; 50 μL of PBS in colour reagent served as blank. The concentration was calculated using the molar absorptivity of I_3_^-^ (ε = 2.46 x 10^4^ M^-1^cm^-1^). Calibration curves obtained with different peroxides such as H_2_O_2_, t-butyl hydroperoxide, and cumene hydroperoxide gave values for ε of 2.45 ± 0.04, 2.34 ± 0.26, and 1,26 ± 0.15 x 10^4^ M^-1^cm^-1^, respectively. A stoichiometric relationship (slope = 1.02) was observed between the amount of organic peroxides assayed and the concentration of I_3_^-^ produced.

### Determination of REM

The electrophoretic runs were performed on agarose gel (1%) and the lipoproteins were precipitated on the gel with phosphotungstate-Mg^2+^ reagent. Electrophoresis was performed in 0.05 M barbital buffer at 100 V for 50 min. REM was defined as the ratio of the migration distance of oxLDL to that of nLDL.

### Determination of oxidation-specific immune epitopes

The formation of oxidation-induced epitopes on apoB was recorded with monoclonal antibodies raised against modified apoB by means of a solid phase dissociation-enhanced lanthanide fluorescence immunoassay (DELFIA^®^) as described previously [8]. Anti-apoB (a rabbit polyclonal antibody purchased from Behring (Marburg, Germany)) and anti-ox-apoB (OB/04) (a monoclonal antibody raised against copper-oxidized LDL which was characterized to react specifically with oxidized apoB-containing lipoproteins, [8]) were used as detecting antibodies. After washing the plates three times, Eu^3+^-labeled rabbit anti-mouse IgG (for OB/04) or Eu^3+^-labeled sheep anti-rabbit IgG (for anti-apoB) were used as reporting antibodies by incubation for 1 h at 25°C. After washing and addition of the enhancement solution (Wallac Oy), fluorescence was measured.

### Malondialdehyde analysis

MDA was determined according to a previously described HPLC method after derivatization with 2,4-dinitrophenylhydrazine (DNPH) [15]. For alkaline hydrolysis of protein-bound MDA 25 μL of 6 mol/L sodium hydroxide was added to 0.125 mL of each fraction obtained at 2 h, 4 h, 6 h, as well as 8 h of Cu^2+^-induced nLDL oxidation (in 1.5 mL tubes) and incubated at 60°C for 30 min in an Eppendorf heater. The hydrolyzed samples were deproteinized with 62.5 μL 35% (v/v) perchloric acid and after centrifugation (14,000 g; 2 min) 125 μL of the supernatant were mixed with 12.5 μL DNPH solution and incubated for 10 min. This reaction mixture, diluted derivatized standard solutions (0.625–10.000 nmol/mL), and reagent blanks were injected into the HPLC system (injection volume: 40 μL). The MDA standard was prepared as previously described [16]. The DNPH derivatives (hydrazones) were separated isocratically on a 5 μm ODS hypersil column (150 x 4.6 mm) guarded by a 5 μm ODS hypersil column (10 x 4.6 mm; Uniguard holder; Thermo Electron Corporation, Cheshire, UK) with a mobile phase consisting of a 0.2% (v/v) acetic acid solution (bidistilled water) containing 50% acetonitrile (v/v). The HPLC separations were performed with an L-2200 autosampler, L-2130 HTA pump, and L-2450 diode array detector (all: VWR Hitachi, Vienna, Austria). Detector signals (absorbance at 310 nm) were recorded and the EZchrom Elite software (VWR International) was used for data acquisition and analysis.

### Cell culture

EA.hy926 cells were obtained from the American type culture collection (ATCC) and were a kind gift of Dr. C.J.S. Edgell (University of North Carolina, Chapel Hill, NC, USA) [17].

EA.hy926 is a permanent cell line that was established by fusing primary human umbilical vein cells with a thioguanine-resistant clone of the human A549 cell line [17]. EA.hy926 cells display characteristic features of primary endothelial cells such as the expression of von Willebrand factor (Factor VIII-related antigen) and synthesis of Weibel-Palade bodies, the exhibition of angiogenesis [18], and the involvement in coagulation, fibrinolysis [19], and inflammation [20].

EA.hy926 cells were cultured in 75 cm^2^ flasks in Dulbecco’s modified Eagle’s medium (DMEM) containing 1 g/L glucose, 3.97 mM L-glutamine, and 1 mM sodium pyruvate supplemented with 10% (v/v) fetal calf serum (FCS), 100 U/mL penicillin, 100 μg/mL streptomycin, and 1x HAT supplement (v/v) at 37°C (under a 5% CO_2_ atmosphere). The split ratio of cells was 1:3–1:4 and only passages below 40 were used for experiments.

For serum starvation (performed overnight) and during cell culture experiments EA.hy926 cells were incubated in serum-free DMEM containing 1 g/L glucose, 3.97 mM L-glutamine, and 1 mM sodium pyruvate supplemented with 100 U/mL penicillin and 100 μg/mL streptomycin at 37°C, 5% CO_2_. nLDL and Cu^2+^-oxidized LDL (oxLDL) were prepared as 1.5 mg/mL stock solutions in PBS and were applied to EA.hy926 cells at a concentration of 0.3 mg/mL for the indicated time periods in serum-free culture medium.

In some experiments serum-starved cells were preincubated with ethyl pyruvate (EP) at concentrations of 250, 500, and 1000 μg/mL (final concentrations) for 45 min at 37°C before addition of oxLDL.

### Electrical cell-substrate impedance sensing (ECIS)

To investigate the effects of nLDL and Cu^2+^-oxidized LDL on EA.hy926 barrier function and monolayer integrity, impedance monitoring was performed using an ECIS Z system (Applied Biophysics, troy, NY, USA). EA.hy926 cells were plated on gold microelectrodes of 8W10E+ arrays, grown to confluence, and then treated with the respective lipoproteins (in the absence or presence of EP) at concentrations of 0.3 mg/ml. After baseline stabilization (20 min after addition of the lipoproteins) impedance was recorded in real time at 4 kHz (barrier function) and 64 kHz (monolayer integrity) for 22 h.

### Cell viability (MTT test)

Cellular viability was assayed using the MTT assay which measures the metabolic activity of living cells. Cells plated in 12 or 24 well plates were grown to confluence and then treated with the respective lipoproteins (in the absence or presence of EP) at concentrations of 0.3 mg/mL for the indicated time periods. MTT (1.2 mM in serum-free medium) was added to cells and incubated for 2 h at 37°C under standard conditions. Cells were washed with PBS and cell lysis was performed with isopropanol/1 M HCl (24:1; v/v) on a microplate shaker at 1200 rpm for 15 min. Absorbance was measured at 570 nm on a Power Wave X Select microplate spectrophotometer (BioTek Germany, Bad Friedrichshall, Germany) and corrected for background absorption (650 nm).

### Measurement of superoxide by HPLC

Cells plated in 12 or 24 well plates were grown to confluence and then treated with the respective lipoproteins (in the absence or presence of EP) at concentrations of 0.3 mg/mL for the indicated time periods. After the incubation time, the medium was aspirated and the cells were washed with HBSS containing 100 μM diethylenetriaminepentaacetic acid (HBSS/DTPA). All subsequent steps concerning addition of DHE and extraction of 2-hydroxyethidium (2-OH-E^+^) have in principle been described by Laurindo et al. [21]. In brief: DHE (50 μmol/L DHE in HBSS/DTPA, freshly prepared) was added to each well and the plates were incubated at 37°C in the dark for 30 min. DHE was aspirated and cells were washed with HBSS/DTPA. One mL of trypsin solution (1:10 dilution in PBS) was then added to the cells and the plates were incubated at 37°C for 5 min. After detaching the cells were transferred into 1.5 mL Eppendorf tubes. All subsequent steps were performed on ice. The cell suspension was centrifuged at 1500 rpm at 4°C for 5 min. The supernatant was discarded and 200 μL of acetonitril was added to the cell pellets for lysis with additional treatment by vortexing and ultra-sonication in a cooled ultrasonic bath for approx. 10 min. After centrifugation at 12,000 rpm at 4°C for 10 min the supernatant was dried under vacuum. The pellets after acetonitrile extraction were dissolved in 200 μL of NaOH (0.1 mol/L) for determination of protein concentration using the BCA Protein Assay (Pierce). Dried supernatants (stored at -20°C up to 2 weeks) were dissolved in PBS/DTPA prior to HPLC analysis.

HPLC analysis was performed in principle according to a method described by Zielonka et al. [11]. In brief, separation of DHE-derived products was performed on a Kromasil column (5 μm, 250 mm × 4.6 mm I.D. equipped with a 10 x 4 mm precolumn) using an L-2200 autosampler and two L-2130 HTA pumps. A linear gradient from 10% acetonitrile to 70% acetonitrile (containing 0.1% trifluoroacetic acid (TFA) as well as 0.1% TFA in the aqueous solution) was performed at a flow rate of 0.7 mL/min within 46 min. Injection volume was 40 μL. Detection of the DHE-derived products was performed with an L-2480 fluorescent detector (DHE: excitation 358 nm, emission 440 nm; 2-OH-E^+^ and E^+^: excitation 510 nm, emission 595 nm) and an L-2450 diode array detector (all: VWR Hitachi)). Detector signals were recorded and the EZchrom Elite software (VWR International) was used for data requisition and analysis.

### Measurement of the cellular high energy content by HPLC

Cells plated in 12 or 24 well plates were grown to confluence and then treated with the respective lipoproteins (in the absence or presence of EP) at concentrations of 0.3 mg/mL for the indicated time periods. After the incubation time the cells were washed with PBS, trysinized, and transferred into 1.5 mL Eppendorf tubes. After centrifugation the supernatant was discarded and the cells were deproteinized with 200 μL of 0.4 mol/L perchloric acid. After centrifugation 150 μL of the acid extract was neutralized with 15–20 μL of 2 mol/L potassium carbonate (4°C). The supernatant obtained after centrifugation was used for HPLC analysis (injection volume: 40 μL). The pellets of the acid extracts were dissolved in 0.5–1.0 mL of 0.1 mol/L sodium hydroxide solution and used for protein determination using the BCA assay. The HPLC analytical method for separation of the high energy phosphates has been reported previously [22]. Alterations in brief: Separation was performed on a Hypersil ODS column (5 μm, 250 mm x 4 mm I.D.) using a L-2200 autosampler, two L-2130 HTA pumps, and a L-2450 diode array detector (all: VWR Hitachi). Detector signals (absorbance at 214 nm and 254 nm) were recorded with a personal computer by means of EZchrom Elite software (Scientific Software Inc., San Ramon, CA USA). In addition to the obtained nucleotide concentrations energy charge (EC) of the cells was calculated employing the following formula: EC = (ATP + 0.5 ADP)/(AMP + ADP + ATP).

### Inverted light microscopy

Cells plated in 12 well plates were grown to confluence and then treated with the respective lipoproteins (in the absence or presence of EP) at concentrations of 0.3 mg/mL for the indicated time periods. After washing with serum-free culture medium cells were applied to inverted light microscopy. Images were acquired on an Olympus IX70 Inverted System Microscope (Olympus Optical Co, Hamburg, Germany) equipped with an Olympus FireWire CVIII camera (Olympus Soft Imaging Solutions GmbH, Muenster, Germany). The Cell^B^ software (Olympus Soft Imaging Solutions GmbH) was used for data acquisition and processing.

### Statistics

One-way ANOVA and Bonferroni post tests were used for statistical evaluation of the effects of increasing amounts of EP on indicators of LDL oxidation, cell viability, superoxide anion, and ATP levels. Correlations between EP concentrations and REM were calculated by Pearson’s coefficient of correlation. Statistical significance was set at p < 0.05. *… p ≤ 0.05, **… p ≤ 0.01, ***… p ≤ 0.001.

## Results

### EP inhibits nLDL oxidation induced by Cu^2+^ ions in vitro concentration-dependently

**Fig 1:**
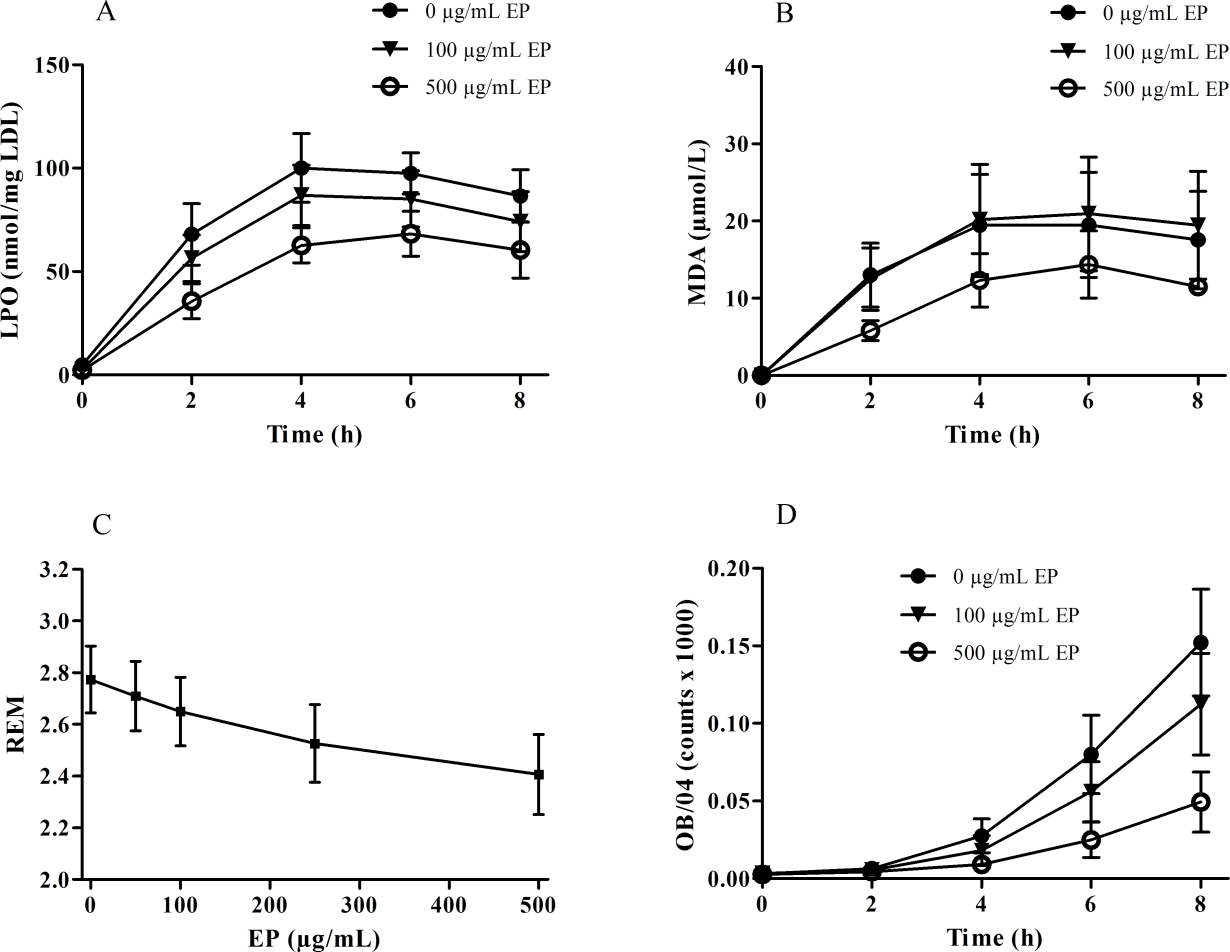
Effect of increasing amounts of EP on nLDL oxidation induced by Cu^2+^ ions in vitro. nLDL (1.5 mg/mL) was preincubated in the absence or presence of EP (100 and 500 μg/mL) and then oxidized by addition of 10 μmol/L CuCl_2_ for up to 8 h. **(A)** EP significantly decreased LPO formation (p < 0.05), **(B)** MDA formation (p < 0.05), and **(D)** the amount of oxidation-specific epitopes (p < 0.05). **(C)** REM concentration-dependently decreased when LDL was oxidized in the presence of increasing amounts of EP (p < 0.005). Data represent mean ± SD from three separate experiments.

EP efficiently suppressed the oxidation of the lipid part of the LDL particle. Formation of LPOs as well as of MDA were found to be significantly decreased when nLDL was oxidized in the presence of increasing amounts of EP (Fig 1A and Fig 1B). Correspondingly, EP also suppressed the oxidative modification of apoB, the protein part of the LDL particle: REM values as well as the amount of oxidation-specific epitopes were concentration-dependently decreased when nLDL was oxidized in the presence of increasing amounts of EP (Fig 1C and Fig 1D).

### The cytotoxicity of oxLDL in EA.hy926 cells is decreased when formed in the presence of EP

**Fig 2:**
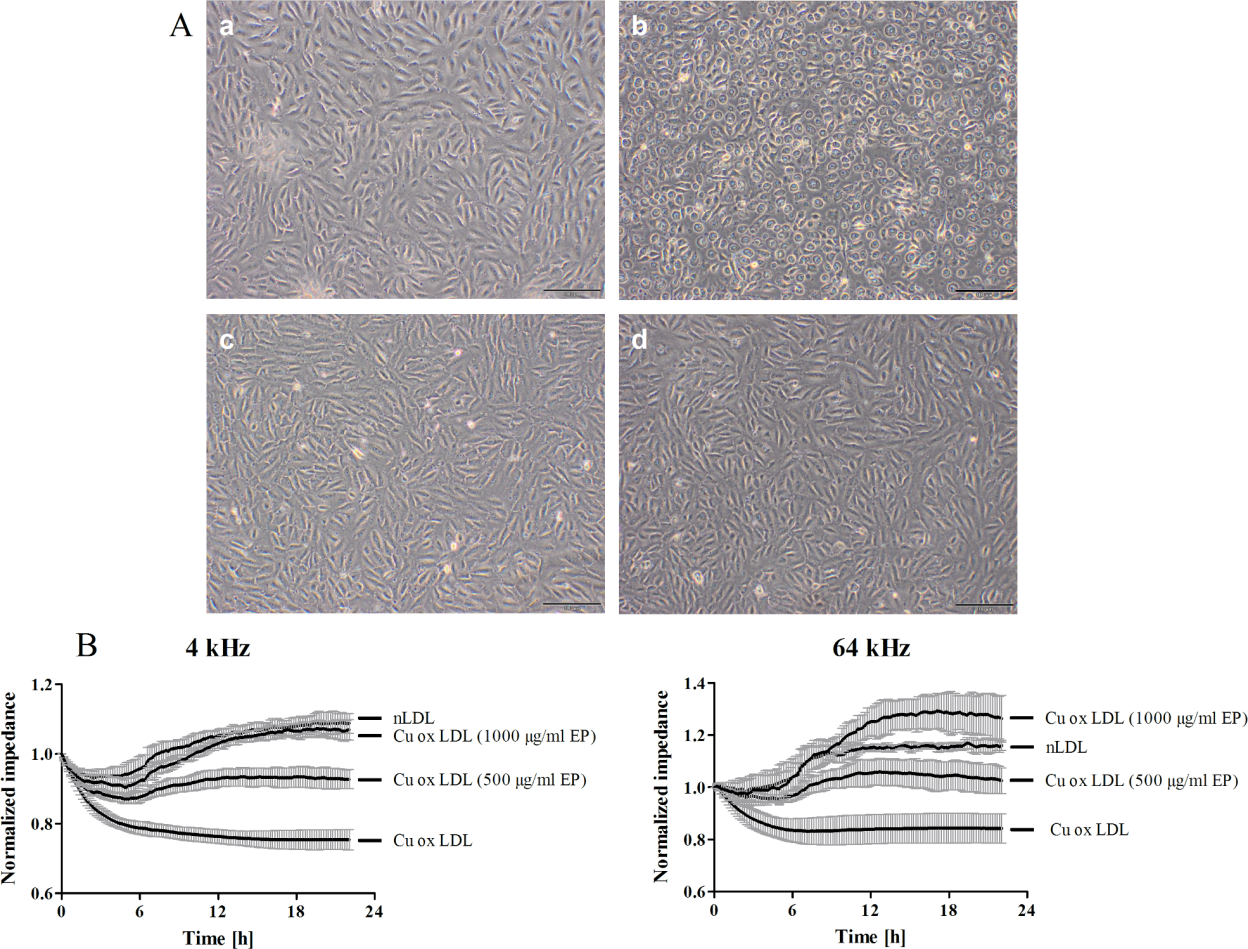
LDL oxidized in the presence of EP does not impact on cell monolayer integrity and barrier function of EA.hy926 cells. **(A)** EA.hy926 cells grown on 12 well plates were treated with 0.3 mg/mL of either nLDL (a), oxLDL (b), or LDL which was oxidized in the presence of EP (500 μg/mL (c) and 1000 μg/mL (d)) for 4 h. After washing with serum-free culture medium cells were subjected to inverted light microscopy. **(B)** EA.hy926 cells grown on gold microelectrodes were treated as described above. Impedance was continuously monitored at 4 and 64 kHz starting after baseline stabilization (20 min post treatment start). Data represent mean ± SD (n = 4).

Microscopic imaging showed loss of monolayer integrity and cell detachment when EA.hy926 cells were treated with oxLDL, formed in the absence of EP (highly oxidized LDL) compared to cells treated with nLDL (Fig 2A, panel a vs. panel b) which is indicative for cellular dysfunction and suggests cell death. The presence of 500 and 1000 μg/mL EP during LDL oxidation mitigated the cytotoxic effects of oxLDL which then did not impact on the EA.hy926 monolayer (Fig 2A, panels c and d). To investigate monolayer integrity and barrier function in more detail ECIS measurements were performed. Results revealed that ox-LDL treatment reduced both, barrier function and monolayer integrity of EA.hy926 cells (Fig 2B). LDL oxidized in the presence of 500 μg/ml EP was almost without effect on barrier function and monolayer integrity and LDL oxidized in the presence of 1000 μg/ml EP as well as nLDL even slightly increased both parameters.

**Fig 3:**
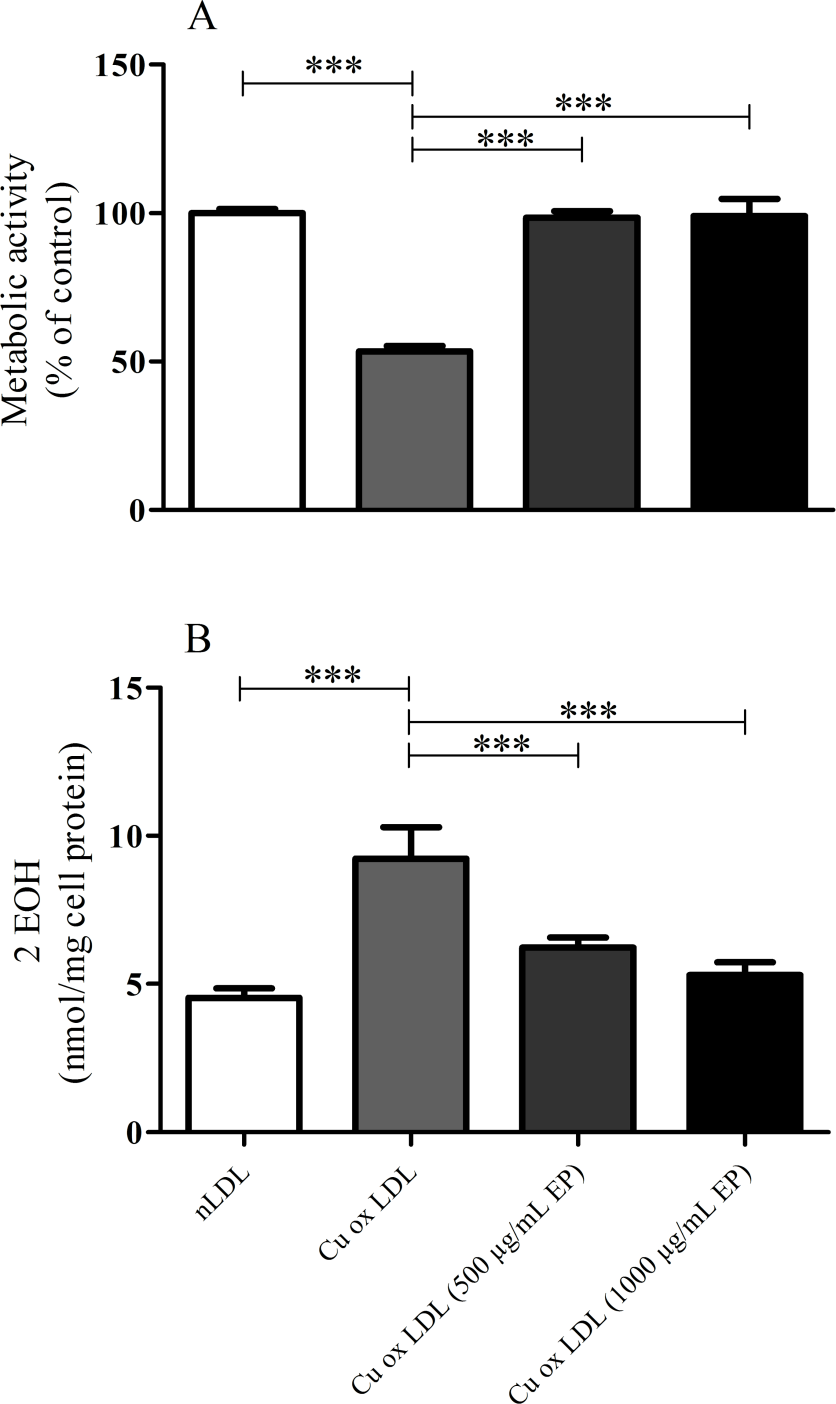
Effect of increasing amounts of EP on the cytotoxic effects of Cu^2+^-oxidized LDLs in EA.hy926 cells. nLDL (1.5 mg/mL) was preincubated in the absence or presence of 500 or 1000 μg/mL EP and then oxidized by addition of 10 μmol/L CuCl_2_ till LPO levels reached 100 nmol/mg LDL protein. Subsequently, EA.hy926 cells were incubated with the thus obtained oxLDLs. **(A)** Cell viability concentration-dependently increased and **(B)** intracellular superoxide radical anion formation concentration-dependently decreased with oxLDLs obtained under increasing levels of EP. Data represent mean ± SD (n = 7), *** p < 0.001.

To investigate cellular viability in more detail the MTT assay was used. Results obtained indicated a significantly reduced viability of EA.hy926 cells treated with highly oxidized LDL (53% of nLDL treatment; Fig 3A). Concomitantly, intracellular superoxide levels were elevated more than 2-fold (Fig 3B), indicating massive oxidative stress in oxLDL-treated EA.hy926 cells. When oxidation of nLDL was performed in the presence of EP (500 or 1000 μg/mL, respectively), the cytotoxicity of the thus formed oxLDLs decreased, reflected in restored cell viability and superoxide levels which were comparable to cells treated with nLDL (Fig 3A and Fig 3B).

**Fig 4:**
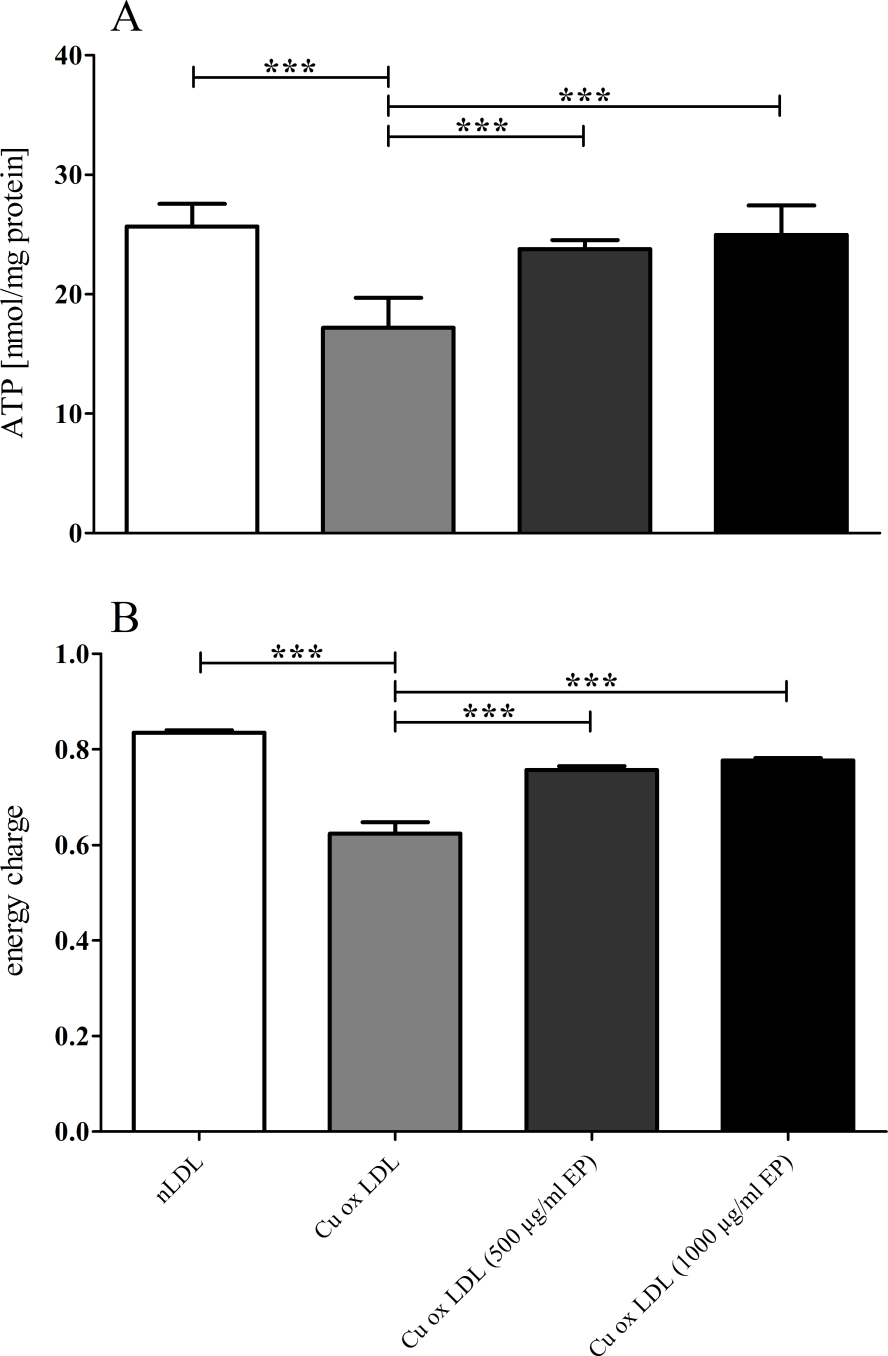
ATP levels and EC in EA.hy926 cells treated with highly or mildly oxidized LDL. EP concentration-dependently attenuated the cytotoxic effect of Cu^2+^-oxidized LDL demonstrated by the increase of ATP **(A)** and EC **(B)** in EA.hy926 cells. Data represent mean ± SD (n = 6); ** p < 0.05, *** p < 0.001.

In good agreement, measurements of intracellular high energy phosphates also revealed increased mitochondrial function in cells that were treated with oxLDLs where EP was present during LDL oxidation (Fig 4A and 4B).

### Increasing concentrations of EP in the culture medium reduce the cytotoxic effects of highly oxidized LDL in EA.hy926 cells

nLDL was oxidized by means of Cu^2+^ ions in the absence of EP, resulting in formation of highly cytotoxic oxLDL as shown above. Subsequently, EA.hy926 cells were incubated with this highly oxidized form of LDL when EP (250, 500, or 1000 μg/mL, respectively) was present in the culture medium.

**Fig 5:**
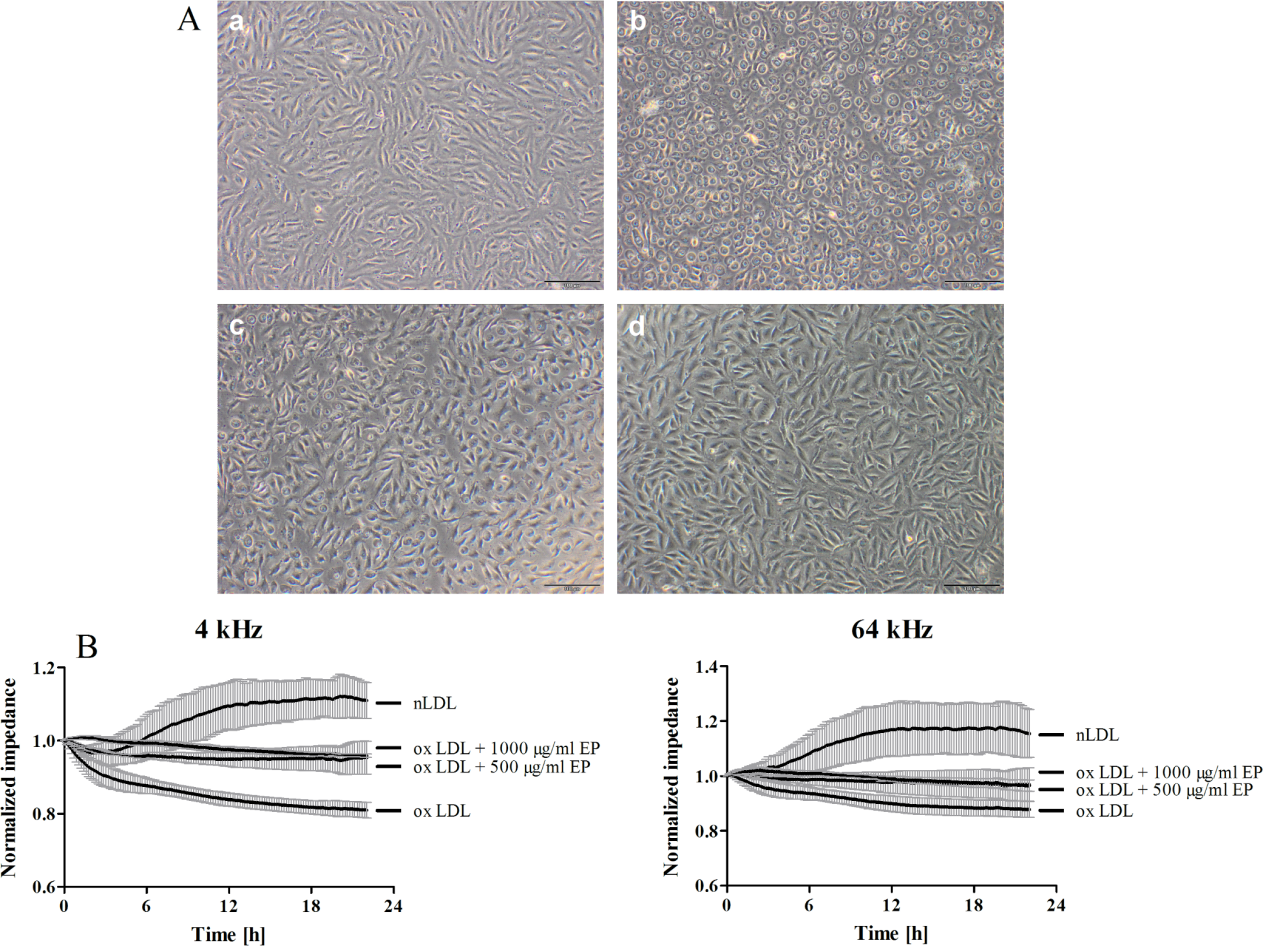
EP restores monolayer integrity and barrier function of oxLDL-treated EA.hy926 cells. **(A)** EA.hy926 cells grown on 12 well plates were treated with 0.3 mg/mL of either nLDL (a) or oxLDL in the absence (b) or presence of EP (c (500 μg/mL) and d (1000 μg/mL)) for 4 h. After washing with serum-free medium cells were subjected to inverted light microscopy. Preincubation of cells with EP was performed for 45 min before addition of oxLDL. **(B)** EA.hy926 cells grown on gold microelectrodes were treated as described above. Impedance was continuously monitored at 4 and 64 kHz starting after baseline stabilization (20 min post treatment start). Data represent mean ± SD (n = 4).

In contrast to an intact cell monolayer of nLDL-treated EA.hy926 cells microscopic imaging again revealed loss of monolayer integrity and cell detachment of cells treated with highly oxidized LDL with EP absent in the culture medium (Fig 5A, panel a vs. panel b). The presence of 500 μg/mL EP in the culture medium almost completely prevented cell detachment although it did not preserve an intact EA.hy926 monolayer (Fig 5A, panel c). In contrast, the presence of 1000 μg/mL EP completely restored monolayer integrity (Fig 5A, panel d). Results of ECIS measurements showed that EP at concentrations of 500 and 1000 μg/ml prevented barrier dysfunction and monolayer disintegration of EA.hy926 cell treated with ox-LDL when compared to cells that were incubated in the absence of EP (Fig 5B).

**Fig 6:**
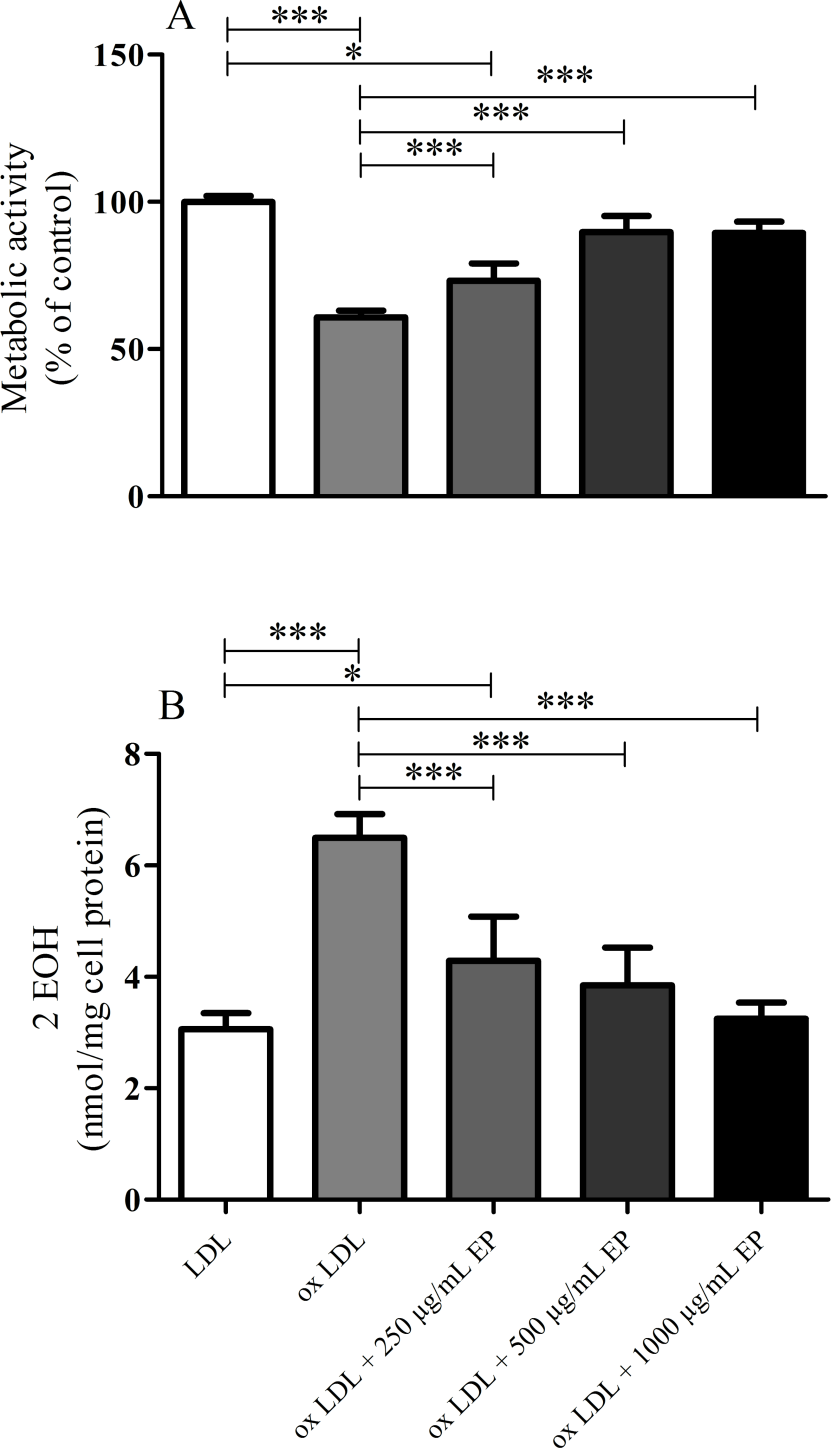
Cytotoxic effects of highly oxidized LDL on EA.hy926 cells in the presence of increasing medium concentrations of EP. nLDL (1.5 mg/mL) was oxidized by addition of 10 μmol/L CuCl_2_ in the absence of EP in order to obtain the highly cytotoxic form of oxLDL. EP added to the culture medium concentration-dependently attenuated the cytotoxic effect of this oxLDL on EA.hy926 cells, reflected in increased viability **(A)** and decreased levels of superoxide radical anion formation **(B)**. Data represent mean ± SD (n = 6 for metabolic activity, n = 4 for superoxide levels), * p < 0.05, *** p < 0.001.

Correspondingly, the viability of oxLDL-treated cells increased (Fig 6A) and superoxide radical anion formation decreased (Fig 6B) the higher the concentration of EP in the culture medium during oxLDL treatment.

**Fig 7:**
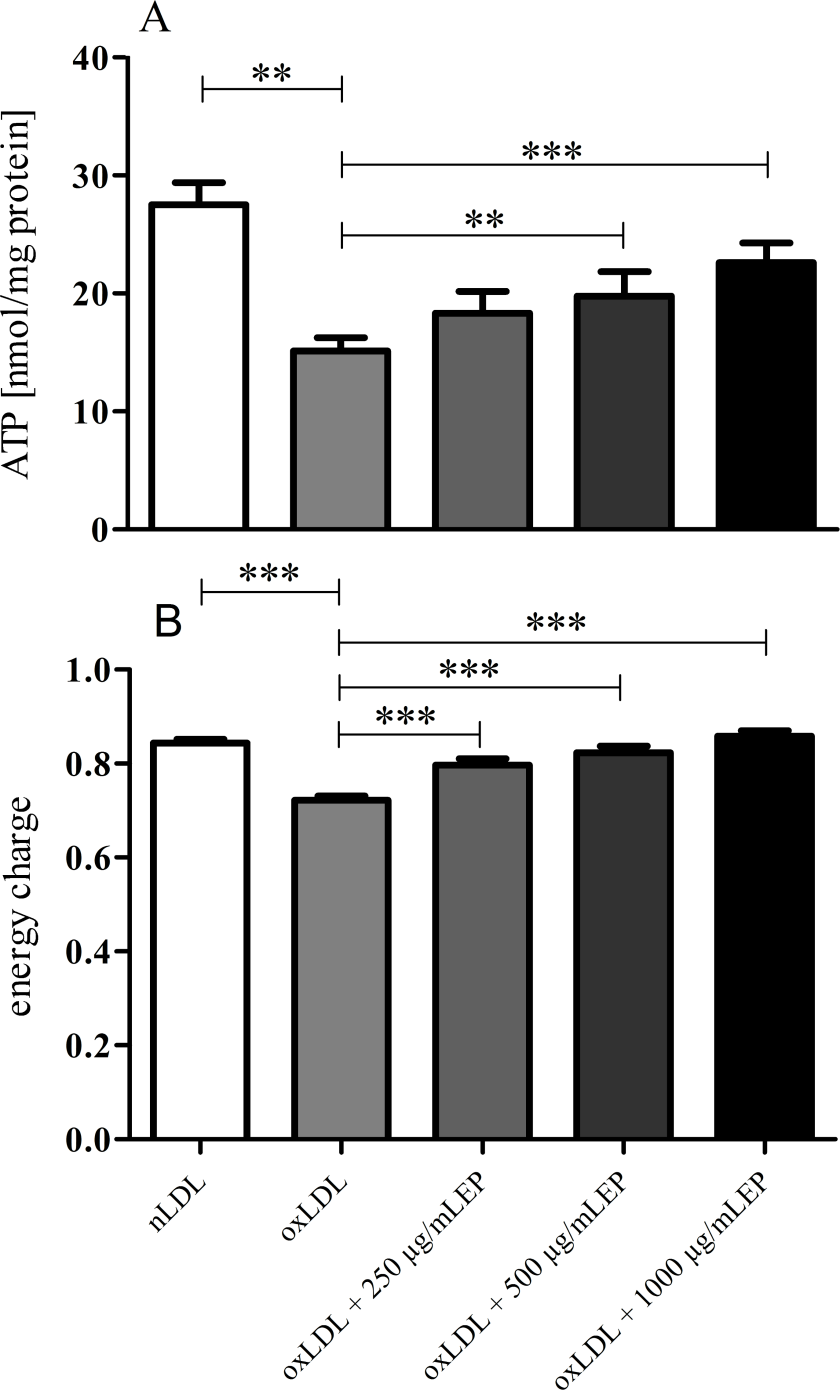
ATP levels and EC in EA.hy926 cells treated with highly oxidized LDL in the presence of increasing medium concentrations of EP. EP concentration-dependently attenuated the cytotoxic effects of highly Cu^2+^-oxidized LDL demonstrated by the increase of ATP levels **(A)** and EC **(B)** in EA.hy926 cells. Data represent mean ± SD (n = 6), ** p < 0.05, *** p < 0.001.

In addition, measurements of intracellular high energy phosphates also revealed that augmented EP concentrations in the culture medium increased ATP levels in EA.hy926 cells. At a concentration of 500 or 1000 μg/mL of EP this increase in intracellular ATP became highly significant (Fig 7A). The calculated energy charge revealed a significant difference already at the lowest level of supplementation with EP (Fig 7B).

## Discussion

In the present study we show that EP is a potentially anti-atherosclerotic drug due to two modes of action. Mode 1: EP is capable of attenuating the oxidation of nLDL mediated by Cu^2+^ ions; Mode 2: EP is capable of directly attenuating the cytotoxic effects of oxLDL in EA.hy926 cells. The EA.hy926 cell line used in this study is a fusion line between human umbilical vein endothelial cells and the A549 lung cancer cell line. Although it might be argued that this cell line is not representative of vascular EC (immortalization, genetic instability) this fusion hybrid has many conserved functions of endothelial cells such as expression of von Willebrand factor (Factor VIII-related antigen) and synthesis of Weibel-Palade bodies, the exhibition of angiogenesis [18], and the involvement in coagulation, fibrinolysis [19], and inflammation [20].

Regarding mode 1: Significantly lower levels of LPO, MDA, REM, and oxidation-specific epitopes as well as a decrease of cytotoxicity on EA.hy926 cells were found when nLDL was oxidized in the presence of increasing amounts of EP. To our knowledge, the underlying mechanisms by which EP attenuates oxidation of nLDL have not been investigated so far.

Elucidation of the mechanisms by which EP attenuates Cu^2+^-mediated LDL oxidation is difficult since the chemical mechanisms by which copper ions oxidize LDL in vitro are still somewhat elusive. Binding of Cu^2+^ ions to the LDL particle and subsequent reduction of Cu^2+^ to Cu^+^ appears to be a pivotal step [23]. Involvement of preformed lipid hydroperoxides in this reduction has been suggested [24, 25] and an accelerating effect of α-tocopherol has been assumed [26]. However, Cu^+^ ions are probably not in contact with the polyunsaturated fatty acids (PUFAs) inside the LDL particle and, therefore, cannot initiate lipid peroxidation directly.

In aqueous solutions, it is known that Azobis(2-amidinopropane) dihydrochloride (AAPH) decomposes to amidinopropyl radicals, which, in the presence of oxygen, are rapidly converted into hydroperoxyl radicals [27, 28]. Therefore, we oxidized nLDL with AAPH in the presence of increasing amounts of EP in a separate set of experiments. These data are presented as supplemental information (S1 and S2 Figs). We also found an attenuation of the AAPH-induced oxidation of nLDL by EP. Thus, this attenuated AAPH-mediated oxidation of nLDL in the presence of increasing amounts of EP gives rise to the suspicion that EP is capable of scavenging hydroperoxyl radicals, a mechanism which presumably also plays a role in the Cu^2+^-mediated oxidation of LDL.

An alternative pathway might be that Cu^+^, bound on the surface of the LDL particle, is capable of generating superoxide, as shown by Burkitt and Duncan [29]. EP has been shown to be capable of scavenging superoxide [30]. This might be, at least in part, another underlying mechanism by which EP is capable of attenuating the Cu^2+^-mediated oxidation of nLDL.

In addition, Burkitt and Duncan have shown that superoxide radical anion formation during Cu^2+^-mediated oxidation of nLDL subsequently leads to formation of hydroxyl radicals [29]. The rapid rate of reaction between the hydroxyl radical and the biomolecules on the surface of the LDL particle does not allow the hydroxyl radical to diffuse into the LDL particle more than 1 or 2 molecular diameters. Thus, hydroxyl radicals are very unlikely the initiators of the peroxidation of PUFAs in the core of the LDL particle.

However, it has been shown that superoxide, under physiological conditions, partially becomes protonated to yield the hydroperoxyl radical [31]. The hydroperoxyl radical is the most stable lipid radical formed in vitro. Due to its uncharged nature, it is very likely capable to diffuse to the lipid rich region in the core of the LDL particle and initiate peroxidation of PUFAs. Results from our AAPH-mediated LDL oxidation experiments suggest that EP is an efficient scavenger of hydroperoxyl radicals. This may be an additional mechanism by which EP is capable of attenuating the Cu^2+^-mediated oxidation of nLDL, when hydroperoxyl radicals are formed. It cannot be ruled out that EP, after permeating the lipid rich region of LDL due to its lipophilic properties [32], might scavenge additional radicals generated during Cu^2+^-mediated oxidation of nLDL.

Regarding mode 2: The protective effect of EP against oxLDL-induced EA.hy926 cell injuries is apparently attributable to its superoxide anion scavenging qualities. It is well known that oxLDL induces oxidative stress in vascular endothelial cells via multiple pathways. For example, oxLDL stimulates endothelial NADPH oxidase, leading to enhanced formation of superoxide, a potent initiator of cell proliferation [33, 34]. Moreover, oxLDL causes uncoupling of endothelial NOS (eNOS), also leading to formation of superoxide rather than NO [35]. Our data clearly show that EP attenuates oxLDL-induced superoxide formation in EA.hy926 cells in a concentration-dependent manner, thereby enhancing cell viability.

Results from numerous recent studies suggest that EP might be a pluripotent pharmacological agent due to its anti-inflammatory, anti-coagulant, and anti-oxidative properties. In various animal models of critical illness treatment with EP has been shown to improve survival and/or amelioration of organ dysfunction, e.g. I/R injury in stroke [16, 36], I/R injury in an electrical burn model [37], brain injury [38], myocardial I/R injury [39], and whole body radiation-induced injury [40]. Anti-inflammatory and anti-oxidative action of EP has also been observed in models using human cells, e.g., in cultured Caco-2 transformed human intestinal epithelial cells [41], in HUVECs [42], in A549 human transformed pulmonary epithelial cells [43], and in A549 human alveolar epithelial cells [44]. Furthermore, EP administration has been shown to inhibit cancer growth in human gastric adenocarcinoma tissues [45].

However, disappointing results were obtained so far when EP was administered to human patients for short-term treatment. In a clinical trial of patients undergoing cardiac surgery, EP failed to improve the outcome, i.e. the inflammatory reactions usually accompanying this surgical treatment [46].

On the other hand, when administered over a prolonged period of time, EP is apparently capable of exerting anti-oxidative action. For example, chronic administration of EP has been shown to be capable of suppressing inflammatory bowel disease or to slow the rate of growth of malignant tumors [47, 48].

With respect to the results of the present study, we suggest that chronic administration of EP might be a suitable tool to attenuate the formation of atherosclerotic plaques. Our data clearly indicate that EP is capable of attenuating the oxidation of both the lipid as well as the protein part of LDL, early and crucial steps in atherogenesis [1]. Our suggestion is supported by the findings of Xiao et al. who have recently reported that EP attenuates high mobility group box-B1 (HMGB-1) expression in mice macrophages. This attenuated expression is associated with a reduced atherosclerotic lesion size in vivo [49]. Moreover, EP has been shown to be capable of reducing vascular endothelial inflammation, an important mechanism involved in atherogenesis, by attenuating endoplasmatic reticulum stress [50].

## Supporting information

S1 Fig Effects of increasing amounts of EP on nLDL oxidation induced by AAPH in vitro. nLDL (1.5 mg/mL) was preincubated in the absence or presence of EP (500 and 1000 μg/mL) and then oxidized by addition of 10 mmol/L AAPH till LPO levels exceeded 100 nmol/mg LDL protein. **(A)** Representative microscopic images of EA.hy926 cells treated with highly or mildly AAPH-oxidized LDL. a) EA.hy926 cells incubated with nLDL; b) EA.hy926 cells incubated with AAPH-oxidized LDL which was formed in the absence of EP (= highly oxidized LDL); c) EA.hy926 cells incubated with AAPH-oxidized LDL which was formed in the presence of 500 μg/mL EP; d) EA.hy 926 cells incubated with oxLDL which was formed in the presence of 1000 μg/mL EP (= mildly oxidized LDL). **(B)** Cell viability was restored to nLDL values when AAPH oxidation was performed in the presence of EP. Data represent mean ± SD (n = 4), *** p < 0.001.
(PDF)

S2 Fig Cytotoxic effects of highly AAPH-oxidized LDL on EA.hy926 cells in the presence of increasing medium concentrations of EP. nLDL (1.5 mg/mL) was oxidized by addition of 10 mmol/L AAPH in the absence of EP in order to obtain highly cytotoxic form of oxLDL. EP added to the culture media concentration-dependently attenuated the cytotoxic effect of this AAPH-oxidized LDL in EA.hy926 cells. **(A)** a) EA.hy926 cells incubated with nLDL; b) EA.hy926 cells incubated with highly AAPH-oxidized LDL in the absence of EP in the culture medium; c) EA.hy926 cells incubated with highly AAPH-oxidized LDL in the presence of 500 μg/mL EP in the culture medium; d) EA.hy926 cells incubated with highly AAPH-oxidized LDL in the presence of 1000 μg/mL EP in the culture medium. **(B)** Cell viability of AAPH-oxidized LDL-treated EA.hy926 cells concentration-dependently increased with EP present in the culture medium. Data represent mean ± SD (n = 4), * p < 0.05, *** p < 0.001.
(PDF)

S1 File Data availability.xlsx. (XLSX)

## Abbreviations

AAPH: Azobis(2-amidinopropane) dihydrochloride
ApoB: apolipoprotein B
BCA: bicinchoninic acid
DELFIA: dissociation-enhanced lanthanide fluorescence immunoassay
DHE: dihydroethidium
DMEM: Dulbecco’s modified Eagle’s medium
DNPH: 2,4-dinitrophenylhydrazine
DTPA: diethylenetriaminepentaacetic acid
EA.hy926: a human vascular endothelial cell line
EC: energy charge
ECIS: electrical cell-substrate impedance sensing
EDTA: ethylenediaminetetraacetic acid
EP: ethyl pyruvate
Eu: europium
FCS: fetal calf serum
HMGB-1: high mobility group box-B1
HNE: 4-hydroxynonenale
HPLC: high-performance liquid chromatography
2-OH-E^+^: 2-hydroxyethidium
IgG: immunoglobulin G
LDL: low-density lipoprotein
LPO: lipid hydroperoxides
MDA: malondialdehyde
MTT: 3-(4,5-dimethyl-2-thiazolyl)-2,5-diphenyl-2H-tetrazolium bromide
OB/04: monoclonal antibody raised against copper-oxidized LDL
oxLDL: oxidatively modified LDL
PUFAs: polyunsaturated fatty acids
REM: relative electrophoretic mobility
TFA: trifluoroacetic acid
