# Supplementary figures and images for: Ethyl pyruvate inhibits oxidation of LDL in vitro and attenuates oxLDL toxicity in EA.hy926 cells
Source: PLoS One. 2018 Jan 25;13(1):e0191477. doi: 10.1371/journal.pone.0191477 (PMC5784938; doi:10.1371/journal.pone.0191477)

Suppl. Fig 1

A

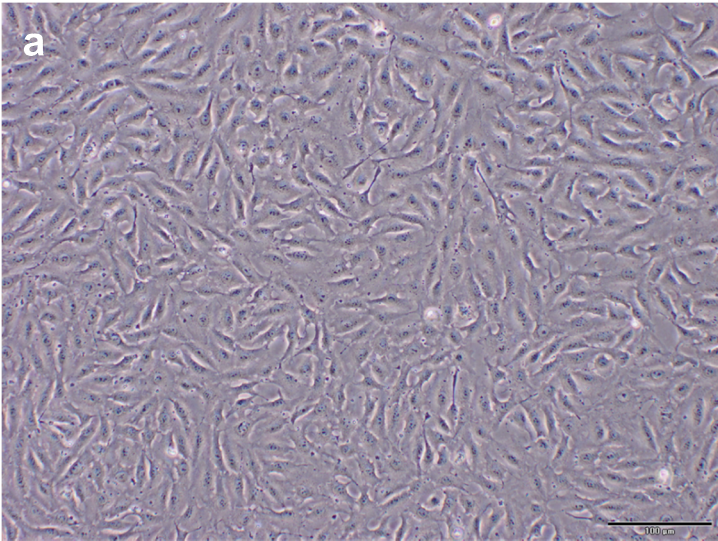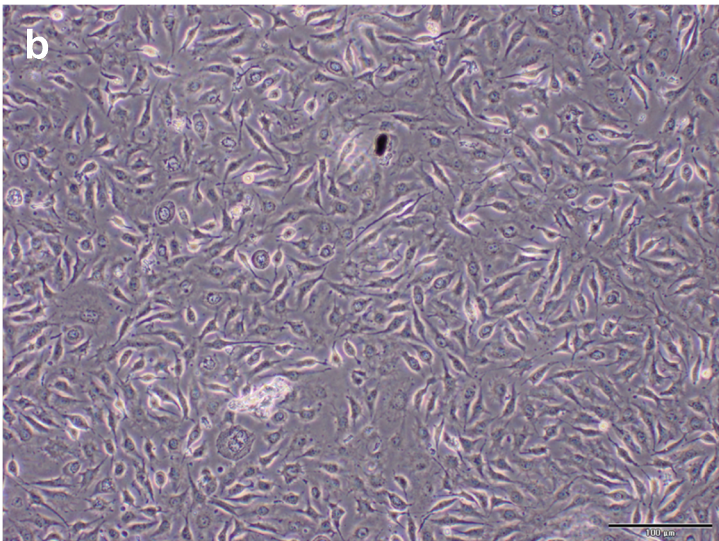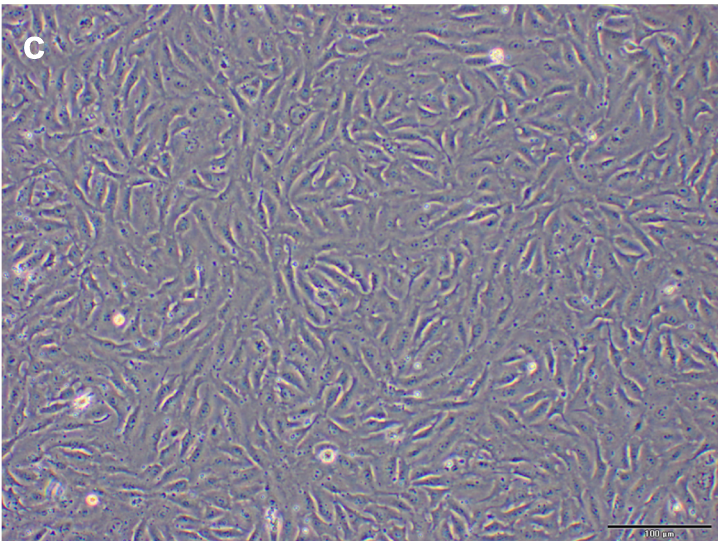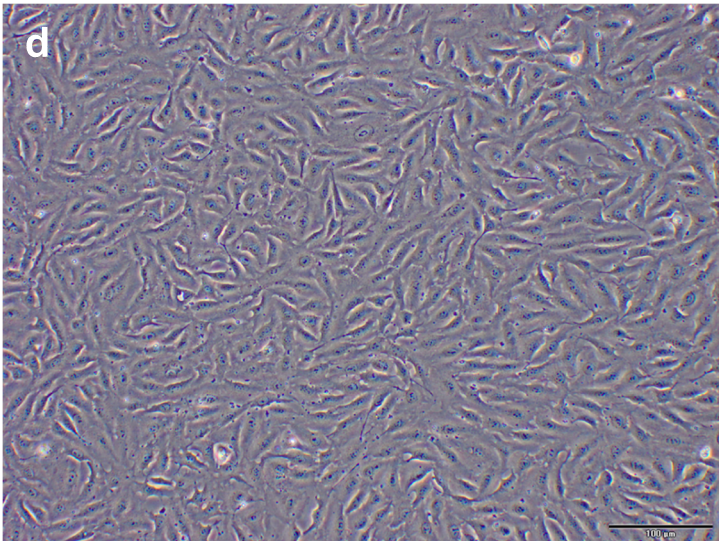

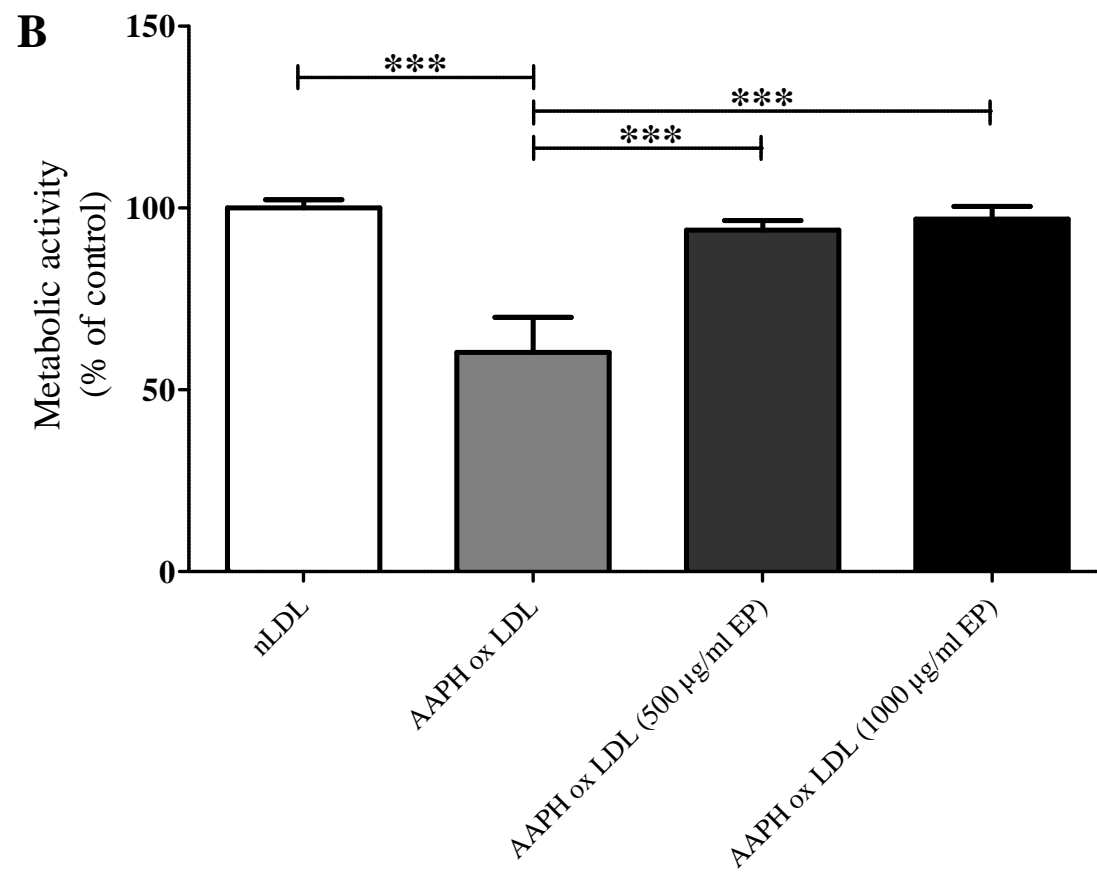

Supplement: S1 Fig — nLDL (1.5 mg/mL) was preincubated in the absence or presence of EP (500 and 1000 μg/mL) and then oxidized by addition of 10 mmol/L AAPH till LPO levels exceeded 100 nmol/mg LDL protein. (A) Representative microscopic images of EA.hy926 cells treated with highly or mildly AAPH-oxidized LDL. a) EA.hy926 cells incubated with nLDL; b) EA.hy926 cells incubated with AAPH-oxidized LDL which was formed in the absence of EP (= highly oxidized LDL); c) EA.hy926 cells incubated with AAPH-oxidized LDL which was formed in the presence of 500 μg/mL EP; d) EA.hy 926 cells incubated with oxLDL which was formed in the presence of 1000 μg/mL EP (= mildly oxidized LDL). (B) Cell viability was restored to nLDL values when AAPH oxidation was performed in the presence of EP. Data represent mean ± SD (n = 4), *** p < 0.001. (PDF) [file pone.0191477.s001.pdf]

Suppl. Fig 2

A

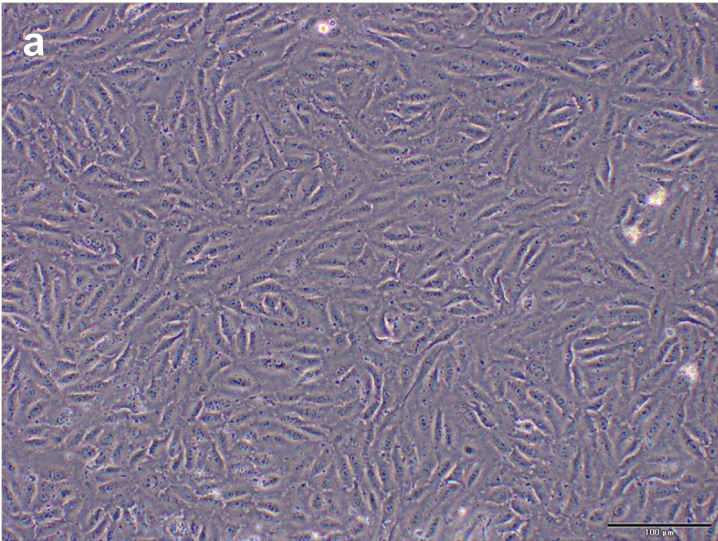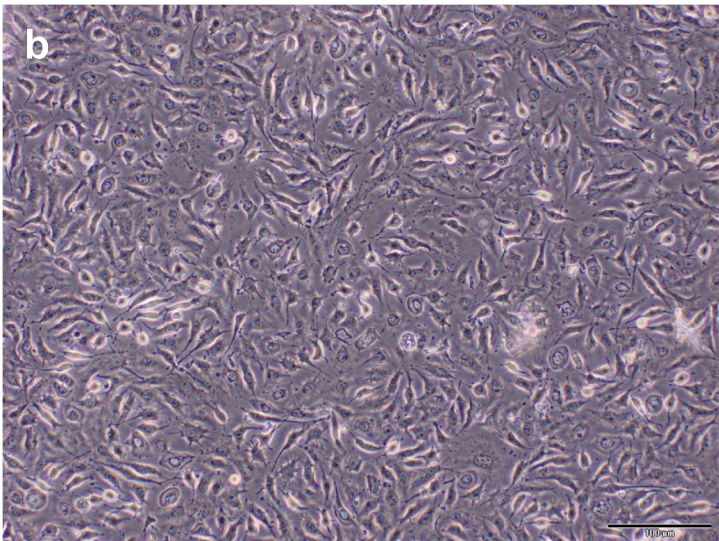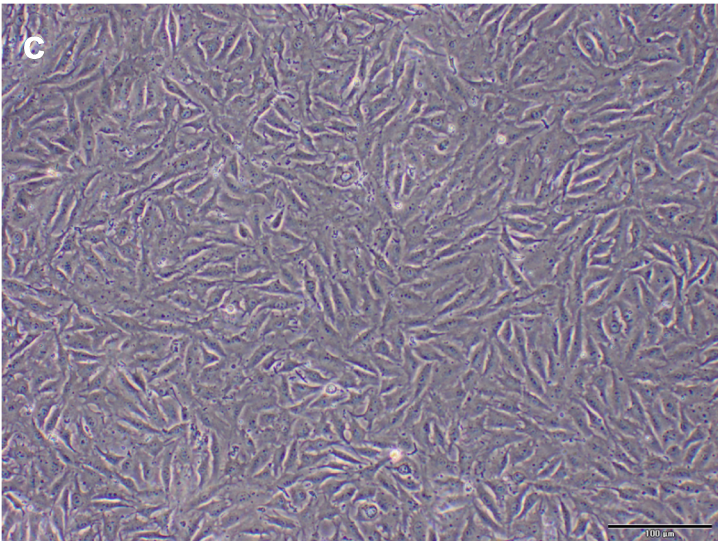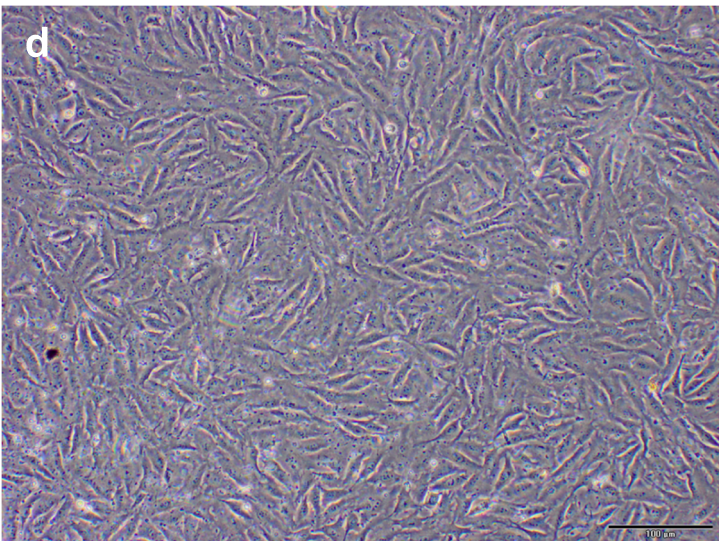

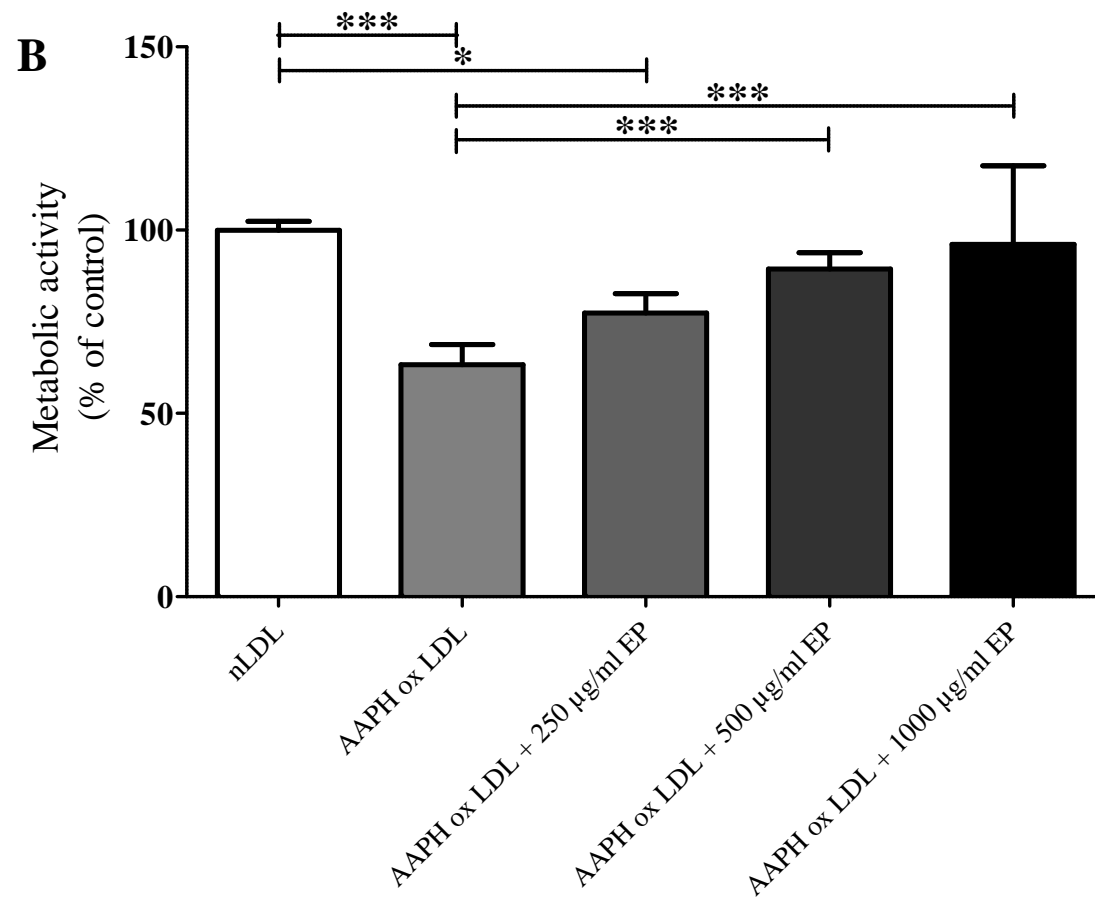

Supplement: S2 Fig — nLDL (1.5 mg/mL) was oxidized by addition of 10 mmol/L AAPH in the absence of EP in order to obtain highly cytotoxic form of oxLDL. EP added to the culture media concentration-dependently attenuated the cytotoxic effect of this AAPH-oxidized LDL in EA.hy926 cells. (A) a) EA.hy926 cells incubated with nLDL; b) EA.hy926 cells incubated with highly AAPH-oxidized LDL in the absence of EP in the culture medium; c) EA.hy926 cells incubated with highly AAPH-oxidized LDL in the presence of 500 μg/mL EP in the culture medium; d) EA.hy926 cells incubated with highly AAPH-oxidized LDL in the presence of 1000 μg/mL EP in the culture medium. (B) Cell viability of AAPH-oxidized LDL-treated EA.hy926 cells concentration-dependently increased with EP present in the culture medium. Data represent mean ± SD (n = 4), * p < 0.05, *** p < 0.001. (PDF) [file pone.0191477.s002.pdf]
